# The effect of repetition- and scenario-based repetition strategies on anatomy course achievement, classroom engagement and online learning attitude

**DOI:** 10.1186/s12909-022-03564-8

**Published:** 2022-06-24

**Authors:** Mehmet Ali Çan, Çetin Toraman

**Affiliations:** 1 Faculty of Medicine, Department of Anatomy, Çanakkale Onsekiz Mart University, Çanakkale, Turkey; 2 Faculty of Medicine, Department of Medical Education, Çanakkale Onsekiz Mart University, AD 17020 Çanakkale, Turkey

**Keywords:** Repetition drill, Anatomy education, Achievement, Online learning attitude

## Abstract

Anatomy is known to be the oldest and most fundamental branch among medical sciences. That is the reason why it is given at the beginning of medical education to form the basis for other medical sciences. Students who newly begin medical education need to spare plenty of time outside the course hours to study Anatomy which involves different and a lot of terminology. In this study, online repetitions were done outside the class using the repetition (classical presentation) and scenario-based repetition methods and the knowledge levels, course engagement statuses and online learning attitudes of the students were compared quantitatively and qualitatively between the groups.

The study was conducted with 162 medical school year 2 students. These 162 students were randomized to experimental and control groups. The data were obtained with “Anatomy Achievement Test (AAT)”, “Classroom Engagement Inventory (CEI)” and “Medical School Students’ Attitudes Towards Online Learning Scale (MSSATOLS)”. After administering the experimental procedure to the students who were randomized to the experimental and control groups, focus group interviews were held with 16 students from the experimental group, 8 who received the highest scores and 8 who received the lowest scores from the data collecting instruments. The collected research data determined that the affective engagement (AE) and the anatomy achievement test (AAT) performed pre- and post-study were higher in the group in which the scenario-based repetition strategy was applied. AAT pre-test (mean = 27.16) and post-test (mean = 27.15) scores of the repetition group were very close to each other. However, the AAT post-test (mean = 32.33) average of the scenario-based repetition group was above the mean pre-test scores (mean = 26.79) (*p* < .05). Similarly, the mean AE pre-test (mean = 17.79) and post-test (mean = 17.91) scores of only the repetition group were very close to one another. However, the AE post-test (mean = 19.46) mean score of the scenario-based repetition group was above the mean pre-test score (mean = 17.82) (*p* < .05). In summary, pre-test and post-test scores changed the anatomy achievement and affective engagement scores, and this change was in favour of experimental group and increasing the post-test scores.

The responses given to the questions in the scales and the impressions obtained from qualitative interviews indicated that the students did not find adequate the lectures given in the form of presentations alone and thought that various methods and primarily scenario-based education should be used as part of anatomy education to be able to establish a good connection with clinical sciences and Anatomy education should be provided not only at the beginning of the medical education but also in the following years.

## Introduction

When talking about medical education, maybe the branch that comes to mind first is “Anatomy”. This is because the material being worked on is the human body and this structure needs to be known very well before anything else. The changes and differences occurring in this structure can only be easily understood if the body structures are known very well. This is why the basis of medical education since the very early ages has been knowledge of the human body, that is, learning human anatomy. When we look at the medical books that have remained from previous centuries, we see that mostly and invariably information and drawings on the human body have reached our time.

Also in today’s medical education, students start learning the language of medicine at the very beginning of their education owing to Anatomy. The first years of the present medical education are when students begin to learn the language of medicine with the help of Anatomy. Pursuant to the medical education curriculum, the normal structure and functioning of the human body is taught in the first two years with an emphasis on macroscopic anatomy education to form the foundation and then information at cellular level and functioning mechanisms are added on top of this foundation to introduce the topics of basic medical sciences. From the third year of the medical education, differences that may be seen in a normal structure and disorders that may occur rise on this foundation as the contents of clinical medical sciences [1]. Therefore, anatomy education plays a major role in providing sufficient and required level of anatomy knowledge when educating future physicians.

Comprising a large part of medical education, anatomy education is perceived as a burdensome and demanding branch. Within the framework of core education programs widely used in medical education today, parts not linked to clinical practice are being removed from anatomy, as in all medical areas, shortening the time allocated for anatomy education. For this reason, some authors state that the quality of anatomy education has declined increasingly due to reduced education time [2].

Developments in educational sciences necessitated changes in medical education and differences in the methodology of medical education have appeared in recent years. Particularly due to new possibilities made available by developing technology, anatomy education started using novelties as all other areas of medical education. In anatomy education, illustrations and dissections are very important in terms of clinical approaches [3–5]. Since visual materials such as drawings, pictures and videos can be easily used by everyone both inside and outside the educational institutions owing to technological developments, this has undermined cadaver dissections, which once was a must for conventional anatomy education.

The constructivist learning theory states that humans are not active recipients during learning. Learners construct knowledge (such as construction of knowledge in phenomenological philosophical view) from their activities during learning and the meanings they ascribe to the reflections of these activities returning to them. This theory emphasizes a) active involvement of learners in the learning process and b) learners’ creating their own meanings through learning from experiences. The constructivist learning theory prioritizes problem-based learning. It is important to encourage the learner during a learning process. This theory supports peer-learning strategies in a learning environment. Learners are motivated when they have the right to control the contents, strategies and activities of learning [6–8].

Today, many methods are being used in anatomy education. Examples of these include “jigsaw” technique, self-directed learning, problem-based learning (PBL), computer-assisted learning (CAL), and methods using virtual reality [9–11]. Studies have shown that there is no single teaching method superior to the others. Using a combination of various methods seems to provide the most benefit. When traditional teaching consisting of lessons and dissections is used in combination with many other methods such as modelling, imaging, computer assisted learning, problem-based learning via clinical cases, surface anatomy, clinical correlation lessons, peer teaching, team-based learning (TBL), case-based learning (CBL), and flipped classroom, this provides major opportunities for the students that improve and encourage their learning [12–16]. The value of these newly used methods should be assessed in terms of to what extent they help students digest basic knowledge and improve their understanding of the topic [11]. Although many new methods are being implemented, most of the medical school students prefer dissection-based anatomy education carried out on a face-to-face basis [17]. It was found in a study that face-to-face education performed using PowerPoint presentations in the classrooms, which is a widely used method in medical education, was preferred by only 3.9% of the students [16]. Scenario-based learning gives learners the opportunity to be active and to develop their daily living skills [18, 19]. This learning approach fills the gap between theory and practice [20, 21]. When structured in association with daily living, scenarios support learners in their effort to establish a connection with the practices they will encounter in their future professional lives [22]. Gossman et al. have reported that scenarios that have been successfully linked to daily living contribute to effective learning of learners [23]. Learning with scenarios not only serves to effective and realistic learning but also enables learning activities to become more entertaining and the process more satisfactory. Owing to this feature, it also supports motivation [24].

The current Covid-19 pandemic has revealed that education methods to be used from now on should be reviewed again and adapted to changing circumstances [25]. We see today that the integration of the basic knowledge acquired with the clinical sciences has become the criterion for success in medical education. That is why the medical education curriculum is dynamically updated according to the requirements of the day. Use of case discussions, which will establish the connection with clinical sciences that will be studied in the coming years of medical education, is necessary and will undoubtedly increase the quality in anatomy education [26–28].

Studies have shown that giving anatomy education at the first stages of medical education involves some drawbacks. It has been argued that to prevent this, anatomy education supported by modern education methods should be spread over the entire stages of medical education [29, 30]. Whatever the education method used, the largest portion allocated in morphological sciences during the first years of medical education is the learning of anatomy topics, be it in the classroom or laboratory, or at home [31]. During their studies outside the school to learn the topics of anatomy, it is important for the students to have access to modern methods besides their own learning methods they use when reviewing the lesson. Many study materials they can use online during their own studies can be found on both university web pages and social media networks [32]. Although students have a large number of options they can use during going over their lessons, studies have shown that the study material they use most is the lecture notes at hand [33, 34].

### Purpose of the research

The objective of this study is to investigate the effect of topic repetition or scenario-based repetition strategy on achievement, classroom engagement and online learning attitude in medical school students who study anatomy topics outside classroom and to obtain the thoughts of the experimental group students who use scenario-based repetition strategy about this experimental procedure. The following hypotheses will be tested in this study.

***H***_***1***_: There is a significant difference between the pre-test achievement scores of the students who went over their anatomy lessons using the repetition strategy and scenario-based repetition strategy before anatomy classes.

***H***_***2***_: There is a significant difference between the post-test achievement scores (gain levels) of the students who went over their anatomy lessons using the repetition strategy and scenario-based repetition strategy after anatomy classes.

***H***_***3***_: There is a significant difference between the pre-test classroom engagement scores of the students who went over their anatomy lessons using the repetition strategy and scenario-based repetition strategy before anatomy classes.

***H***_***4***_: There is a significant difference between the post-test classroom engagement scores of the students who went over their anatomy lessons using the repetition strategy and scenario-based repetition strategy after anatomy classes.

***H***_***5***_: There is a significant difference between the pre-test online medical education attitude scores of the students who went over their anatomy lessons using the repetition strategy and scenario-based repetition strategy before anatomy classes.

***H***_***6***_: There is a significant difference between the post-test online medical education attitude scores of the students who went over their anatomy lessons using the repetition strategy and scenario-based repetition strategy after anatomy classes.

## Method

This study used a mixed method intervention design which incorporates both quantitative and qualitative data. Owing to collection of more detailed and sufficient data in studies, the mixed method has been developed further and used widely in recent years [35] to identify the strong aspects of quantitative and qualitative data [36] and to consolidate such data [37]. The mixed intervention design allows use of qualitative data to support the quantitative data obtained from an experimental study [38].

**Fig. 1:**
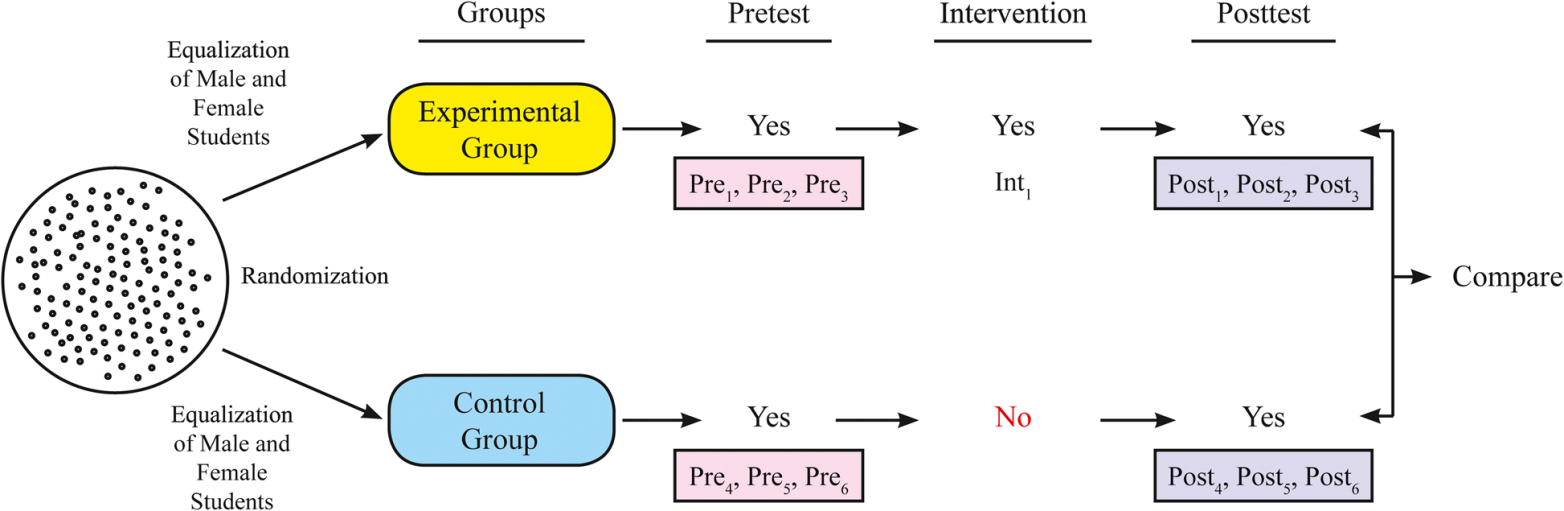
Quantitative part of the research. Pre1: “Anatomy Achievement Test (AAT)” pretest application in the experimental group. Pre2: “Classroom Engagement Inventory (CEI)” pretest application in the experimental group. Pre3: “Medical School Students’ Attitudes Towards Online Learning Scale (MSSATOLS)” pretest application in the experimental group. Pre4: “Anatomy Achievement Test (AAT)” pretest application in the control group. Pre5: “Classroom Engagement Inventory (CEI)” pretest application in the control group. Pre6: “Medical School Students’ Attitudes Towards Online Learning Scale (MSSATOLS)” pretest application in the control group. Int1: Application of “Scenario-Based Repetition Lessons” in the experimental group. Post1: “Anatomy Achievement Test (AAT)” posttest application in the experimental group. Post2: “Classroom Engagement Inventory (CEI)” posttest application in the experimental group. Post3: “Medical School Students’ Attitudes Towards Online Learning Scale (MSSATOLS)” posttest application in the experimental group. Post4: “Anatomy Achievement Test (AAT)” posttest application in the control group. Post5: “Classroom Engagement Inventory (CEI)” posttest application in the control group. Post6: “Medical School Students’ Attitudes Towards Online Learning Scale (MSSATOLS)” posttest application in the control group. P. S.: In the course of the curriculum, the repetition of the lesson, which was taught with the didactic teaching method in the anatomy lesson during the day, was carried out in the control group with the same method

The quantitative dimension of the research is an experimental study randomly assigned with a pre-test post-test control group design. In the pretest posttest control-group design the research participants are randomly assigned to two or more treatment conditions and a pretest is administered, then the treatment conditions are administered and last, the posttest is administered. This is a mixed design because it has a between-subjects (the treatment groups composed of different participants) and a within-subjects factor of "time" (where all participants receive the pretest at time l and the posttest at time 2) [39]. The design of the quantitative part of the research is shown in Fig. 1.

**Fig. 2:**
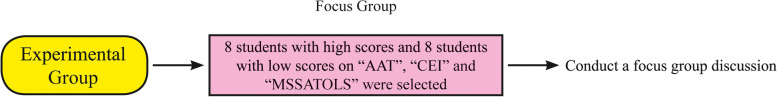
Qualitative part of the research. AAT: Anatomy Achievement Test. CEI: Classroom Engagement Inventory. MSSATOLS: Medical School Students’ Attitudes Towards Online Learning Scale

Focus group interviews were held for the qualitative dimension of the study. The major feature of these focus group interviews was to allow obtaining data in an interactive environment, which could not have been obtained in personal interviews. During group discussions, participants share their views and listen to the views of others, and then they may want to review their own views and express them in a different way [40, 41] or to restructure their own interpretations [42]. The design of the qualitative part of the research is shown in Fig. 2.

### Participants

The study was carried out with Çanakkale University Faculty of Medicine students in the fall semester of 2021–2022 Academic Year. Both the experimental and control groups consisted of 50 female and 31 male medical students. The study was carried out with a total of 162 students. The focus group was formed by including 8 students from the experimental procedure group who had the highest scores and 8 students who had the lowest scores from anatomy achievement, classroom engagement and attitude after the experimental procedure. A large majority of the sampling in qualitative research is purposive sampling. A purposive sample may be chosen according to the maximum variation principle for arguments to be realistic even if it does not meet statistical probability criteria [41]. The number of students in the groups is shown in Table 1.

**Table 1:** The number of students in the groups

Groups	Female	Male	Total
Experimental Group	50	31	81
Control Group	50	31	81
Focus Group	12	4	16

### Data tools

Three different data collecting instruments were used in the study: Anatomy Achievement Test (AAT), Classroom Engagement Inventory (CEI), and Medical School Students’ Attitudes Towards Online Learning Scale (MSSATOLS).

AAT: Developed by the investigators, the test covers anatomy topics taught during two committees (eight weeks) and measures the knowledge level. The test was composed of five-item multiple choice type questions. The test included 50 items. The lowest score obtainable from the test was “0” and the highest “50”. To determine its validity and reliability, the test developed was administered on a trial basis before the study. The test was administered to one upper grade medical school students who had taken the course a year before (year 3, *N* = 100). Before engaging in the experimental procedures, which were the essential procedures of the study, there was a need for an anatomy test with evidenced validity and reliability. The validity and reliability evidences of such a test can be obtained from persons who are knowledgeable of the lesson. For this reason, the first thing that was done before starting the study was to carry out pilot administrations of this scale to one upper grade students (who have already taken the course) and to identify its psychometric characteristics. From the results obtained, item difficulty (pj), item discrimination (rjx) and KR-20 reliability were studied (the validity of multiple-choice test items is determined by the discriminateness and difficulty levels of the questions and their reliability by the KR-20 coefficint). As a result of the analyses, items with an item difficulty between 0.40 and 0.65 and an item discrimination between 0.30 and 0.60 were included in the AAT. The KR-20 reliability coefficient was found to be 0.88. According to Nunnally and Bernstein sufficient reliability should be at least 0.70 and above [43]. The necessary adjustments were made to render the test valid and reliable.

CEI: The Classroom Engagement Inventory was adapted to Turkish by Sever [44]. CEI measures the level of classroom attendance in high school students. The original inventory was developed by Wang, Bergin and Bergin. The inventory, which had 24 items in its original form, was composed of 23 items and 5 subscales after having been adapted to Turkish. The inventory is a five-point Likert type measurement tool. The Turkish version of the inventory was found to have a Cronbach Alpha reliability value of 0.93. According to Nunnally and Bernstein sufficient reliability should be at least 0.70 and above [43]. With a CEI validation factor analysis, the goodness of fit indices were obtained as recommended in the literature. CEI subscales and items included: Affective engagement (items 1–6), behavioural engagement-compliance (items 7–10), behavioural engagement-effortful classroom participation (items 11–13), cognitive engagement (items 14–20), and disengagement (items 21–23).

MSSATOLS: Developed by Yurdal et al. [45], this scale is a measurement tool consisting of 22 items and two subscales. The scale is a five-point Likert type measurement tool. The Cronbach Alpha reliability value of the scale was found to be 0.96. According to Nunnally and Bernstein sufficient reliability should be at least 0.70 and above [43]. With a MISSATOLS validation factor analysis, the goodness of fit indices were obtained as recommended in the literature. The MISSATOLS subscales and their items are: Attitudes towards online learning (items 1–11) and attitudes towards online medical education (items 12–22).

### Anatomy education in Çanakkale Onsekiz Mart University Medical Education curriculum

Anatomy courses are given in year 1 and 2 in Çanakkale Onsekiz Mart University (ÇOMU). Year 1 anatomy courses include “Medical Terminology” in committee 1, “Movement System I” in committee 4, “Movement System II” in committee 5 and “Movement System III” in committee 6. Year 2 anatomy courses include “Circulatory System” in committee 1, “Hemapoietic System and Respiration” in committee 2, “Gastrointestinal System and Metabolism” in committee 3, “Neuroendocrine System I” in committee 4, “Neuroendocrine System II” in committee 5 and “Urogenital System” in committee 6.

### Process

- The period in which the study was to be conducted (year 2) and the course committees were identified.
- The learning objectives of these course committees for anatomy course were identified.
- A specifications table was constructed for the learning objectives and an AAT was developed in line with this table.
- The AAT developing process involved year 3 students. Validity and reliability were determined based on the data obtained.
- Scenario-based repetition lessons were prepared in accordance with the learning objectives.
- Permissions for use were obtained from the scientists who developed the CEI and MISSATOLS measurement tools.
- Ethics committee permission was obtained.
- A semi-structured interview form was prepared for focus group interviews and necessary amendments were made after obtaining specialist views on the form. The interview form was finalized after this procedure.
- An announcement was made to year 2 students about the study, explaining its scope and those who were willing to take part in the study were identified.
- Informed consent forms were obtained from the students who volunteered to participate in the study.
- One hundred-sixty-two volunteering students were assigned to the experimental and control groups randomly (gender balance was observed when assigning them).
- The courses taken online by the students while following the normal daytime program in the pandemic period were not interfered at all.
- Pre-tests were administered to the experimental and control groups.
- Lesson repetitions were provided online to both the experimental and control groups two evenings a week for 12 weeks (24 h in total for both groups).

- The lesson repetitions were conducted over a scenario in the experimental group.
- Example scenario:
- A 12-year-old boy goes to the sea with his friends on a hot summer day. They decide to hold a competition to have fun while swimming. In an area with a depth of about 2 meters, they try to get it out by throwing stones at the bottom of the sea and diving. While the child is about to dive in and pick up the stone, he feels a pain in his right ear. He sees that the pain disappears when he rises above the water, but reappears every time he dives.
- Questions:
  – Does anyone have experience?
  – What could be the source of the problem?
  – Pharynx parts, what are their relations with the environment?
  – What other problems may accompany it?
  – In which situations do the same/similar findings occur?
  –  What should be done to fix it?
  –  Diagnosis: Eustachian dysfunction

- The lesson repetitions were conducted as plain lesson repetitions similar to their daytime studies in the control group.
- After the end of the experimental procedure, the post-tests were administered.
- A focus group interview was held with the experimental group and the study was terminated.

### Epistemological positions of research

In the quantitative part of the study, the cases were examined with the perspective of “positivism”, which brought the history of science to an important position and had a great influence on scientific research. The principle of trying to obtain generalizable data with data collection tools that have been shown to be valid and reliable has been adopted. In the qualitative dimension of the research, the cases were examined with a “hermeneutic” perspective, which accepts that reality is created in a social environment, argues that each phenomenon is meaningful in its own context, and focuses on revealing this meaning. In this perspective, what comes to the fore is not to obtain generalizable data, but to reveal in-depth meanings. Both perspectives have advantages and disadvantages. A way to take advantage of the advantageous aspects of these perspectives and eliminate their disadvantages can be a “pragmatist” approach. This is the reason why this research utilizes both quantitative and qualitative research approaches.

### Data analysis

At the end of the procedure, the data obtained from AAI, CEI and MSSATOLS were transferred to the R program. The R program was preferred for being cost free software. Multivariate analysis of variance (MANOVA) test was used in the analysis. The level of significance was accepted to be *p* < 0.05. One-way MANOVA is a procedure comparing the mean scores of more than one quantitative variable for participants in two or more groups [46]. In this study, AAT gives a single total score, CEI total scores in five subscales and MSSATOLS total scores in two subscales. For this reason, the number of output/dependent variables in this study is more than one. This requires MANOVA test in analysis.

The qualitative data obtained from the focus group interviews were analysed using the inductive content analysis. Stating that there are many ways to categorize in content analysis, Mayring explained that two types of categorizations are dominant: Inductive and deductive analysis [47]. This study used inductive content analysis. In inductive data analysis, data are first encoded. Analysis goes then from codes to categories, to meta categories and to themes. The content analysis was not conducted by the investigators. It was conducted by 3 medical education specialists who had qualitative research experience and had no relationship with the study. The consistency coefficient between the coding of these 3 specialists was calculated with Krippendorff Alpha. The coefficient was calculated to be 0.93, which is an indication of a high level of consistency [48].

### Application of ethical rules in research

This study was approved by Çanakkale Onsekiz Mart University Scientific Research Ethics Committee (No: 2020-YONP-0021 [date:04/12/2020, decision no:06/20]). An informative meeting was held with the students. The purpose and conduct of the study was explained. They were told that the classes held in the school during the day would continue in their own course and that this study would have nothing to do with their achievement in their courses and no additional achievement grades would be given. They were also informed, however, that the lessons continuing in the normal course of the educational program would be assigned as repetition lessons during the study and this would allow them to listen to the lessons again and support their learning. This became a motivation for the students to volunteer for the study. Volunteering students were asked to sign an informed consent. The students were assigned to the repetition or scenario-based repetition groups randomly not based on their own choices.

## Results

The pre- and post-experiment statuses of anatomy achievement, classroom engagement and attitude towards online learning were determined in the experimental and control groups. The output/dependent variables of the study were the score obtained from AAT, the total scores obtained from the five subscales of CEI and the total scores obtained from the two subscales of MSSATOLS. As explained in the data analysis section, it is appropriate to use MANOVA instead of ANOVA when the number of output/dependent variables is two and more. As a consequence of the study design, there are two factors affecting the output/dependent variables. One of these factors is in different groups (experimental and control groups). The second factor is the measurement performed at different times while the time is advancing (pre-test and post-test). Additionally, the interaction between being in different groups and taking measurements at different times needs to be considered. The scores obtained by the groups before and after the experiment were tested with MANOVA. The results are shown in Table 2.

**Table 2:** Pre-test and post-test comparison in experimental and control groups (MANOVA Test)

Effect	F	p	η^2^
Measure	2,122	0,034	0,051
Group	1,919	0,057	0,047
Measure * Group	2,021	0,044	0,049

*Interaction of Measure between Groups

*F* Result of MANOVA Test, *p* Significancy, *η*^2^ Effect Size

According to the results of the analysis, there was no significant difference between the experimental and control groups with respect to anatomy achievement, classroom engagement and online learning attitude (*p* > 0.05); there was significant difference between the pre-test and post-test scores of the experimental and control groups with respect to anatomy achievement, classroom engagement and online learning attitude (*p* < 0.05); and there was significant difference in anatomy achievement, classroom engagement and online learning attitude based on group and measurement interaction (*p* < 0.05).

If, as a result of a MANOVA test, there is a significant difference between 1) being in different groups, 2) measurements taken at different times and 3) group and measurement interaction, separate ANOVA tests should be run for the factors in which significant differences were found and the ANOVA test results are obtained for the factors in which significant differences were found with MANOVA test. The ANOVA test made in this way shows significant differences on the basis of the output/dependent variable. When a significant difference is found in MANOVA, ANOVA is applied individually to the factors to determine at which factor level the significant difference is. Table 3 shows the ANOVA results based on the interaction of measure from the main effects, additionally being in the experimental or control group and the pre-test post-test group (i.e., group and measure) interaction. The effect size of the significant differences obtained was calculated. According to Cohen’s [49] classification, the effect size was small (η^2^ < 0.05).

**Table 3:** ANOVA Results by Factors

Source	Dependent Variable	F	p	η^2^
Measure	Anatomy Achievement Test (AAT)	4,901	0,028	0,015
	Affective Engagement (AE)	6,671	0,010	0,020
	Behavioral Engagement-Compliance (BE–C)	3,744	0,052	0,012
	Behavioral Engagement-Effortfull Classroom Participation (BE-ECP)	1,675	0,196	0,005
	Cognitive Engagement (CE)	1,367	0,243	0,004
	Disengagement (D)	0,051	0,822	0,000
	Attitudes Towards Online Learning (ATOL)	1,091	0,297	0,003
	Attitudes Towards Online Medical Education (ATOME)	0,154	0,695	0,000
Measure * Group	Anatomy Achievement Test (AAT)	4,911	0,028	0,015
	Affective Engagement (AE)	6,650	0,010	0,020
	Behavioral Engagement-Compliance (BE–C)	3,754	0,054	0,012
	Behavioral Engagement-Effortfull Classroom Participation (BE-ECP)	1,681	0,199	0,005
	Cognitive Engagement (CE)	1,360	0,251	0,004
	Disengagement (D)	0,051	0,822	0,000
	Attitudes Towards Online Learning (ATOL)	1,061	0,304	0,003
	Attitudes Towards Online Medical Education (ATOME)	0,052	0,820	0,000

*Interaction of Measure between Groups

*F* Result of MANOVA Test, *p* Significancy, *η*^2^ Effect Size

**Fig. 3:**
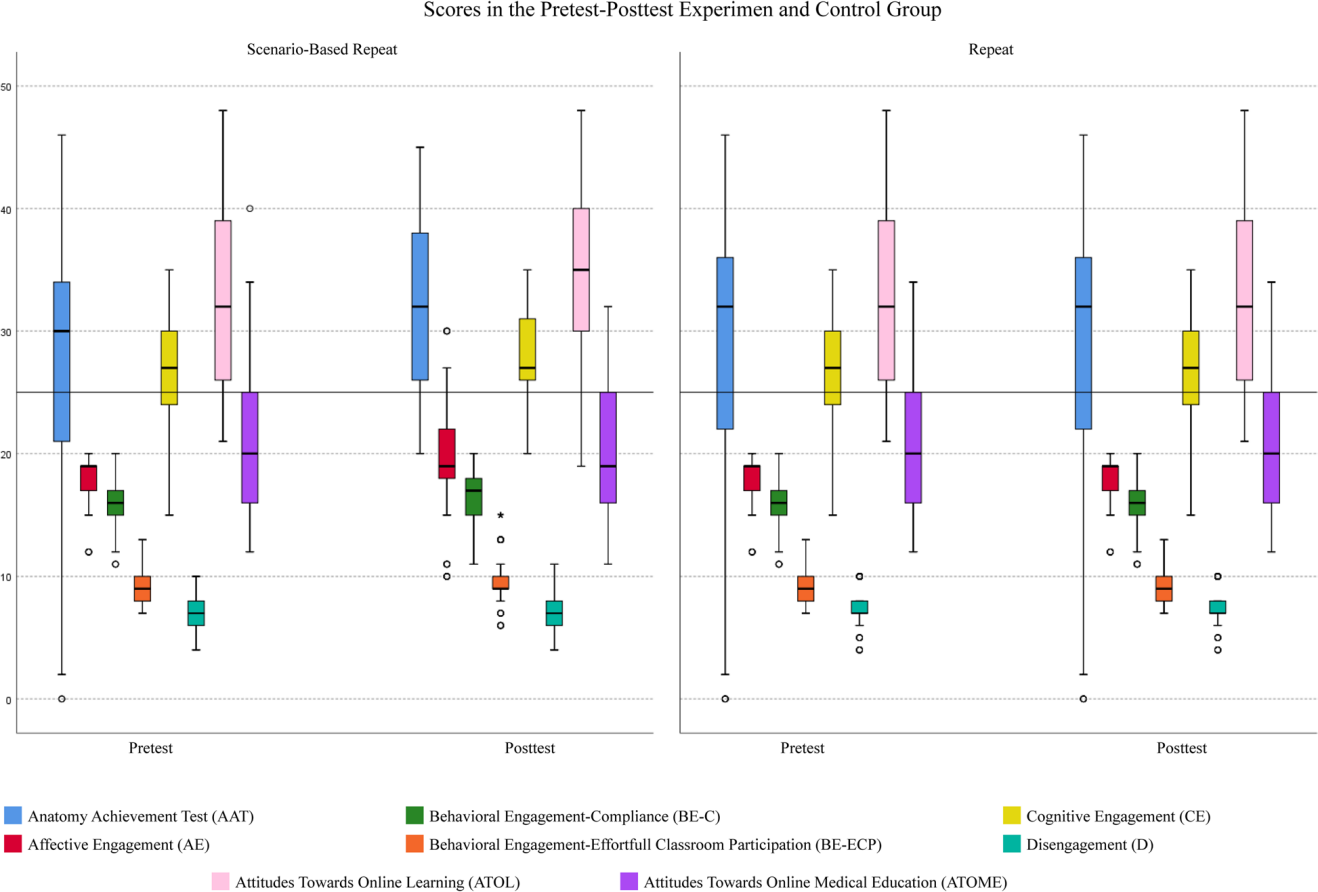
Scores in the pretest–posttest experimental and control group

In addition to the MANOVA test results shown in Table 3, a graphical presentation of the scores obtained is shown in Fig. 3.

It was determined that the measure of the main effects (i.e., pre-test and post-test) made a significant difference on the AAT scores (*p* < 0.05) and the AE sub-dimension of the classroom engagement inventory (*p* < 0.05). The significant difference was found at the small effect size level. There was no significant difference in other scales and subscales (*p* > 0.05).

Measure and group interaction (i.e., being in the experimental or control group with pre-test post-test) on AAT scores (*p* < 0.05) and on the AE sub-dimension of the classroom engagement inventory (*p* < 0.05) was found to create a significant difference. The significant difference occurred at the small effect size level. There was no significant difference in other scales and subscales (*p* > 0.05). ANOVA test is preferred when at least three and more group comparisons will be carried out. If a significant difference is found as a result of the ANOVA test, post-hoc tests are used to determine between which group or groups the significant difference exists.

According to the Bonferroni post-hoc test results applied, the mean AAT pre-test (mean = 27.16) and post-test (mean = 27.15) scores of the repetition group were very close to each other. However, the AAT post-test (mean = 32.33) average of the scenario-based repetition group was above the mean pre-test scores (mean = 26.79). Similarly, the mean AE pre-test (mean = 17.79) and post-test (mean = 17.91) scores of only the repetition group were very close to one another. However, the AE post-test (mean = 19.46) mean score of the scenario-based repetition group was above the mean pre-test score (mean = 17.82). In summary, pre-test and post-test scores changed the anatomy achievement and affective engagement scores, and this change was in favor of experimental group and increasing the post-test scores.

**Fig. 4:**
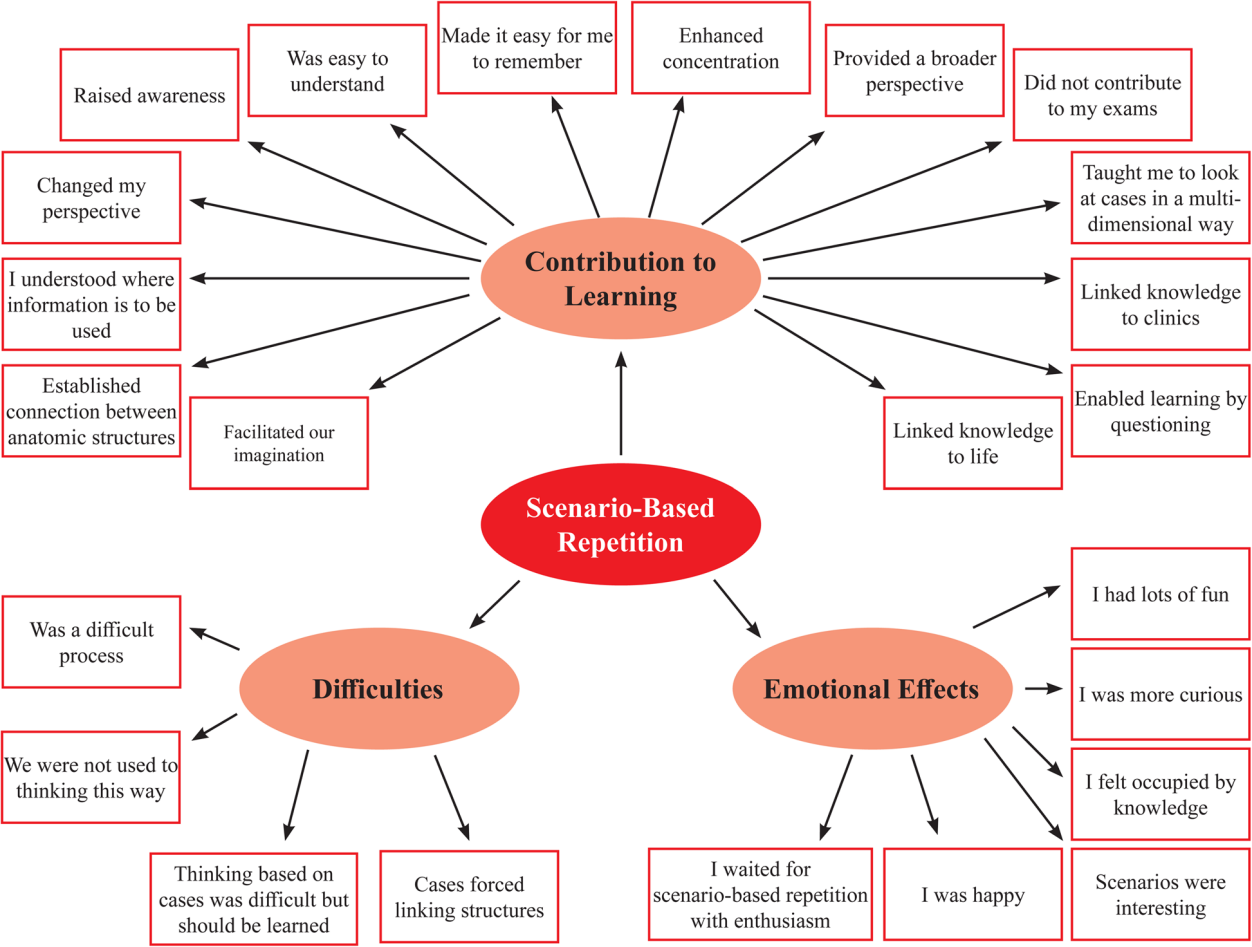
Categories and codes under the scenario-based repetition theme

A focus group interview was held with 16 experimental group students, 8 who had the highest scores and 8 the lowest scores from anatomy achievement, classroom engagement and attitude. The data were analysed with the inductive content analysis and two themes were obtained: Scenario-based repetition and errors made in the anatomy class. The transcript of the interviews showed us that the students evaluated scenario-based repetition with respect to its contribution to learning, its difficulties and its emotional effects. The categories and codes derived from the scenario-based repetition theme are shown in Fig. 4.

The students thought that scenario-based repetition was a method that contributed to learning. It helped improve imagination, interconnecting anatomic structures, discovering the context in which knowledge will be used, developing perspective, awareness, comprehension, remembrance, concentration, multi-dimensional approach, linking knowledge to clinical practice, and questioning. However, it had no effect on their success in examinations. Scenario-based repetition also involved some difficulties. The students were not used to thinking in this way; it was difficult to think over cases and to establish connection between cases and structures. The process had also emotional effects on the students. These emotional effects manifested themselves as happiness, amusement, increased interest and excitement. The following may be given as examples to students’ thoughts and statements after a scenario-based repetition:

*Student 5*: I kind of get drowsy when our professor explains the lesson in our daytime classes. I almost learned to sleep with eyes open, looking! But in scenario repetition classes, our instructor was not telling anything. After explaining the situation, he would ask questions based on the scenario and try to make us find out everything. Only if we could not come to a conclusion he would teach us. It was nice, the scenarios were realistic and questions and answers always kept me awake.

*Student 12*: I had a very hard time in scenario-based repetition classes. I am a year 2 medical school student yet. How little I know! It seemed like the scenarios were telling me; “You are now a physician, this case was brought to you, go ahead and implement what you have learned”. I struggled first, but one gets used to forcing himself to make judgements in time.

**Fig. 5:**
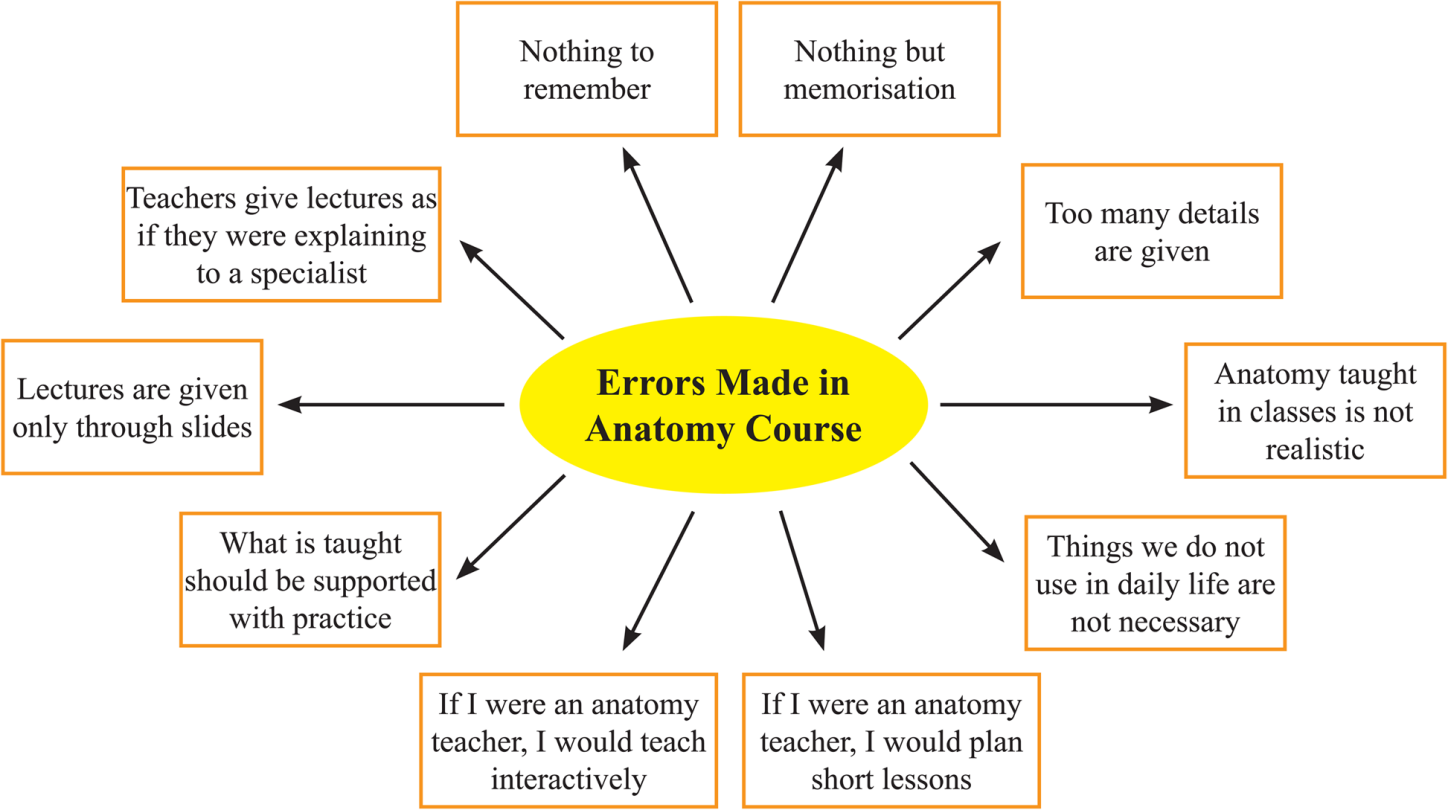
Codes under the errors made in anatomy classes theme

It was found during the interview that the students interpreted the scenario-based repetition method by comparing it with the anatomy classes held at school during the day. This dimension was considered as an ‘errors made in anatomy classes’ theme. The codes derived from the errors made in anatomy classes theme are shown in Fig. 5.

The anatomy lessons provided at the school within the scope of the education program involve a lot of memorizing and cannot achieve retention of information by students, the faculty members lecture as if they lectured to specialists, too many details are given, the lessons are not linked to daily living and are presented only from slides without any interactive teaching and the classes last so long that they become distracting. The following may be given as examples to students’ thoughts and statements after a scenario-based repetition:

*Student 9*: This class I attended showed me how bad the anatomy classes we had at school during the day were. I thought anatomy lessons were not being lectured well at school anyway. Noting but memorization! What they call functional anatomy, the anatomy we’ll need in clinics in the future is not being explained. In my opinion, all anatomy classes should be like how they are in scenario lessons.

*Student 16*: Lecturers are only reading from slides in the classroom. Memorization, memorization, always memorization. Nothing is left in one’s mind! What good would the anatomy lectured in the classroom be to me when I become a physician in the future? It will be enough for me to know how anatomy will appear before me in my profession as in these scenario repetition classes.

## Discussion

The data of the present study showed that the affective classroom engagement of the students who learned the anatomy course in the scenario-based repetition group was higher than the students who learned the anatomy course in the lecture/presentation repetition teaching technique. Consequently, the scenario-based repetition strategy increased the affective participation. We think that the reason for this is that students realize the necessity of this information when it is associated with how they will use the information they have learned in their future life, and they adopt the information by paying more attention so that the information can be permanent. The fact that the students reported that they were satisfied with the scenario-based repetition and gave emotionally positive feedback in the opinions collected from the students in the focus group interview part of the research supports our opinion.

It is an expected result that the engagement rate is higher since scenario-based studies take place as a preliminary experience of professional life. A study made by Singh et al. in 2019 showed that only 3.9% of the students preferred PowerPoint presentations in education [16]. This shows us that students do not favour passive learning methods. In a similar study investigating the contribution of dissection videos used through distance education to anatomy education, attendance was found to decrease gradually in the group that was asked to watch the anatomy videos [50]. Considering the anatomy courses taught in medical education, the difficulty in comprehending the topics that involve different and complicated terminology, which are encountered for the first time in each lesson, is clearly known. Therefore, use of various education methods consisting of consecutive stages for each topic will increase both the interests and subject comprehension levels of students [12, 15].

Many studies have shown that anatomy education combined with clinical case discussions is more effective and medical school students prefer this method more [26–28]. It has been argued that in case-based educations causality is emphasized, it is specified where the knowledge gained will be used and this increases the level of attendance to these educations [12]. In conclusion, the previous studies indicate the necessity of focusing more on the relationship between anatomy courses and other medical fields, especially clinical fields. It has been argued that repetition of reminder information on anatomy during giving clinical information in later years facilitates learning and enables knowledge to be more enduring.

No difference was found between the two groups when the students’ attitudes towards online learning and online medical education were questioned; the low scores obtained showed that the students did not favour online education so much [45]. Considering online medical education based on the data we obtained, we found that, similar to online education, the students did not favour distance medical education and were against online education particularly in the area of practical applications. When we examine the reasons for this situation, we can infer that due to the current pandemic, faculty instructors started education suddenly and using insufficiently developed infrastructure without prior experience, they experienced problems in the online application of formative exams and evaluation methods that measure success that directed students to study regularly and measurement, and students were unable to create group dynamics since they were separated from each other and could not establish a face-to-face relationship. Another negative aspect of students’ being apart from each other relates to the peer education. It is known that students work together and educate each other particularly in laboratory applications. Studies have also underlined the importance of this issue and recommended peer education as an effective method especially when there is a shortage of educators [51, 52]. It has been argued that in peer education the students assuming the role of an educator are better motivated to have access to information so that they can better explain the subject. This is also known by medical education administrators who are trying to develop methods to prevent this. However, any type of method tried seemed to have failed to meet the practical needs of medical education. In an area where visual and three-dimensional comprehension is important as in anatomy, the lack of a substitute for models and cadaver dissections in distance education is overwhelming. The fact that the students were aware of this and revealed it in the scales is very valuable in that it shows they realize the importance of the education they receive. In fact, the values of online medical education are lower than those of online education.

When we compared the results of the pre- and post-applications of the achievement test, which we performed to determine the anatomy achievement level in the pre- and post-study period, it was found out that the scenario-based repetition group showed more improvement. Based on this finding, when we consider that students studying at medical faculties get close scores in the university entrance exam and have a particular study habit, we conclude that the basic sciences information can be better assimilated when associated with clinical situations [53]. Considering that students consciously choose medical faculties and want to start their business life ready in the following years of their lives, it is expected that scenario-based repetition methods will increase interest and perception to a higher level when we think that they work by fulfilling the requirements of education. The point that really matters here is that the students are aware to what end they will learn information and where they will use it.

In the interviews held with the students in the experimental group, we concluded that they favoured the scenario-based repetition method and considered it a method contributing to learning. As contributing factors, they stated that it enhanced imagination, ability to connect anatomic structures with clinical cases, discovering the context in which knowledge will be used, developing perspective, awareness-comprehension-remembrance, concentration, multi-dimensional approach, and questioning. When the emotional effects of this process were questioned, the students stated that they were happy and they had fun, increased interest, curiosity and excitement. However, it was clearly observed in both post-test comparisons with the control group and the medical school’s routine end-of-committee exams that despite all these benefits, it had no contribution to their achievement in exams.

When we questioned the difficulties involved in the scenario-based repetition method, the students stated that they were not used to thinking that way, it was difficult to think of anatomic structures based on cases and it was not easy to establish the link between cases and structures. This method would in fact be difficult when we consider the educator-centred education system widely used in Turkey today. However, considering the clinical phase of the education and the professional life thereafter, the necessity of case presentations where symptomatic approach dominates and developing this type of thinking habits are indispensable.

The conclusion we will draw from this case is that it is not sufficient to conduct only scenario-based research, but also the necessity of using other training and working methods. The same conclusion has also been shown to us with many studies conducted on this subject [12, 13, 15, 16]. The necessity of using the novelties in educational sciences and new education methods emerging today also in medical education is obvious. Today when we can obtain information much more easily compared to the near past, many educators stress the need for using different methods in medical education and conduct studies to try new methods and show their benefits.

It was found during the interview that the students interpreted the scenario-based repetition method by comparing it with the theoretical anatomy classes held at school during the day. It was stated that the anatomy lessons involved a lot of information that should be memorized due to the terms they involve and they cannot achieve retention of the information that needs to be conveyed in the minds of students, the faculty members explained lessons as if they lectured to individuals who had more advanced knowledge, too many details were being given, the information given was not linked to daily living and the lessons did not include interactive teaching methods and were not student-centred because they involved presentations where only the educator was active, and the classes lasted so long that they became distracting. All these supports the necessity of using different education methods in medical education.

In the end-of-committee meeting held with the inclusion of all students in the periods after the termination of the study, the students wanted continuation of practices similar to the scenario-based repetition method that was used during the study and this showed that the entire students, not only those who participated in the study, supported this type of practices. We think that the scenario-based course repetition method, which is an application method they will encounter in the medical profession, is a complementary component of education, in terms of both participating in face-to-face education and understanding the information more easily, in addition to affecting the exam achievement.

## Limitations

When planning the experimental study, it was intended that the experimental group would learn the topics using the scenario-based method and the control group using the conventional teaching method. However, it was not possible to conduct the study in this way. Because the course of education during the day continued on one side and it was not possible to form the groups randomly in education taking place according to the education curriculum on the other side. Additionally, not all the year 2 students volunteered to take part in the study. For these reasons, the experimental design was designed as online repetitions of the lessons taught during the day as per the curriculum in the evening hours. In short, the experimental group repeated the lesson online using the scenario-based repetition and the control group using the conventional teaching methods without disturbing the course of lessons in the curriculum. This is the greatest limitation of the study.

Another limitation is the number of individuals in the experimental and control groups. The study conducted with 81 experimental and 81 control groups can be conducted with larger groups in the future.

Another limitation is the duration of experimental procedure. Planning new studies where the experimental procedure lasts longer, if possible, a full year will reinforce the results obtained from this study.

Using only Anatomy information in scenarios is another limitation. Devising scenarios using more comprehensive information with the inclusion of other branches of science in an integrated system and planning the education accordingly would certainly be more useful for learners. However, this requires allocation of more time and absence of pressure from conventional education on students. A quantitative and qualitative comparison of a group receiving only scenario-based education prepared in an integrated manner and another group receiving only classical education will provide much more beneficial information for shaping medical education.

## Recommendations for the effects of research on practice and students

The conclusion we will draw from this case is that it is not sufficient to conduct only scenario-based research, but also the necessity of using other training and working methods. The same conclusion has also been shown to us with many studies conducted on this subject [12, 13, 15, 16]. The necessity of using the novelties in educational sciences and new education methods emerging today also in medical education is obvious. Today when we can obtain information much more easily compared to the near past, many educators stress the need for using different methods in medical education and conduct studies to try new methods and show their benefits.

The integration of basic and clinical sciences within the medical education curriculum improves the application of basic science principles to clinical decision-making and reinforces students' ability to use the acquired knowledge.

Educational strategies that use contemporary teaching methods and materials by keeping up with technological developments lead to a more efficient education process and easy interaction with the new generation students who adopt and use new technologies.

Humanity may again experience pandemics similar to the COVID-19 pandemic. In this case, again, technology will be the biggest support of education. Methods that activate the learner blended with technology will be among our important tools.

One of the most important outputs of education is achievement. However, it is not the only indicator. There are also many indicators such as engagement in the learning process, being active in this process, and maintaining motivation. Methods that activate the learner and enable them to participate more in the learning processes increase the satisfaction of the learners from the process.

## Data Availability

The datasets used analyzed during the study are available from the corresponding author on reasonable request. The datasets generated during and analyzed during the current study are not publicly available due to (The research data sets generated and analyzed during the current study are not publicly available due to the confidentiality announcement made on the participants but are available from the corresponding author on reasonable and ethical request.) but are available from the corresponding author on reasonable request. We, as authors, hereby confirm that all methods were performed in accordance with the relevant guidelines and regulations stated in Declaration of Helsinki.
